# Functional gait outcomes following derotation osteotomy for transverse plane malalignment in unilateral cerebral palsy

**DOI:** 10.1186/s12887-026-06944-x

**Published:** 2026-05-05

**Authors:** Stefanos Tsitlakidis, Nicholas A Beckmann, Sebastian I Wolf, Paul Mick

**Affiliations:** Clinic for Orthopedics, Heidelberg University Hospital, Schlierbacher Landstr. 200a, Heidelberg, 69118 Germany

**Keywords:** 3D-instrumented gait analysis, Unilateral cerebral palsy, Derotation osteotomy, Lever arm dysfunction, Function, Recurrence

## Abstract

Transverse plane malalignment is a deformity often encountered in patients with Cerebral Palsy (CP) and derotational osteotomy (DO) represents the gold standard for correction of this condition. Reports specifically on unilaterally involved individuals are limited and it remains unclear whether DO is capable to additionally improve hip abductor lever arm dysfunction. 20 individuals with unilateral CP matched the inclusion criteria. Pre- and postoperative (short-term after unilateral DO) 3D-instrumented gait analyses (IGA) were compared and assessed for changes in transverse plane kinematics. Pelvic/trunk kinematics as well as coronal hip kinetics were included. Most remarkable findings were an improved/reduced, yet not physiological, pelvic retraction, significantly improved internal rotation of the hip and significantly improved foot progression. Trunk obliquity worsened postoperatively in the GMFCS level I subgroup. DO effectively improves internal hip rotation and foot progression. There were no relevant changes evident concerning trunk lean, indicating that DO does not relevantly improve hip abductor lever arm dysfunction in unilateral CP at short-term.

## Article summary

- Article focus:
  - Assessing transverse plane improvements, hip abductor lever arm dysfunction and pelvic symmetry following derotation osteotomy (DO) specifically in patients with unilateral CP. These factors are considered to negatively affect the outcome after DO and promote recurrence of transverse plane malalignment.
- Key massages:
  - DO effectively improves internal hip rotation and foot progression
  - There were no relevant changes evident concerning pelvic and hip gait features hip abductor function/trunk lean at short-term
  - Mildly involved individuals (GMFCS level I) even showed clinically relevant worsening of the trunk lean postoperatively.
- Strengths and limitations:
  - First study to exclusively assess mildly and unilaterally involved individuals with motion analysis
  - First study to include trunk, pelvis and coronal hip gait features (besides transverse plane kinematics)
  - Short term follow-up

## Introduction

Transverse plane malalignment defined as an increased internal hip rotation due to an increased femoral torsion – and secondary increased tibial torsion – is a deformity often encountered in patients with Cerebral Palsy (CP) [1, 2]. In principle, internal hip rotation is thought to be compensatory for restoring hip abductor moments that are reduced (lever arm dysfunction) [3] due to the increased femoral torsion, which in turn is caused by unilateral spasticity [4–8]. Additionally, this hip abductor weakness leads to pelvic and trunk obliquity of varying severity as internal hip rotation seems just to partially compensate for the lever arm dysfunction and weak hip abductors [8–12]. In this regard, Trendelenburg and Duchenne limp generally represent sufficient but energy-consuming compensatory mechanisms to unload the weak hip abductors [9, 11, 13–16]. 

Consecutively to the anatomical (increased femoral torsion) and dynamic (internal hip rotation) transverse plane malalignment, as a consequence, even in mildly involved individuals with unilateral CP pelvic retraction is found as a consecutive deviation that works as a compensation mechanism, in order to restore foot progression and thus the forefoot lever arm [3, 4, 17]. Here, the majority of patients with unilateral CP show pelvic retraction [5]. 

Derotation osteotomy (DO) represents the gold standard for correction of transverse plane malalignment [18–20]. In this context, 3D-instrumented gait analysis (IGA) represents the gold standard in the evaluation of motion/gait disorders and plays a key role in preoperative planning and as a tool for control/follow-up evaluation, particularly as the torsional profile and function in the transverse plane might differ significantly [21–27]. 

Reports specifically on unilaterally involved individuals are limited. Most studies report on the effects and limitations of femoral derotation osteotomy (FDO) in patients with bilateral CP due to small numbers of unilaterally involved individuals, mainly for FDO as part of a single event multi-level surgery (SEMLS) and mainly including transverse plan gait features [18, 20, 27, 28]. There are inconsistent reports available, describing recurrence rates up to 40% [29, 30]. Persisting hip abductor lever arm dysfunction and persisting pelvic retraction as well as a younger age, reduced hip joint impulse and internal foot progression angle postoperatively were described to be associated/identified factors for this phenomenon [19, 31, 32]. 

However, it is questionable if obtained findings analyzing bilaterally involved individuals are applicable to unilaterally involved individuals due to the naturally given asymmetry as the most apparent and significant difference. So far, it remains unclear whether DO is capable to additionally and significantly improve lever arm dysfunction, thus improve Duchenne limb and restore pelvic symmetry besides in-kneeing/in-toing in patients with unilateral CP.

Therefore, the objective of this work was a detailed analysis and comparison of preoperative and the postoperative transversal and coronal plane gait features before and following DO specifically in patients with unilateral CP. A further intent was the evaluation and comparison of patients with different motor ability as there might be relevant and specific differences in gait features between different levels of functional impairment.

## Patients & methods

This work was conducted as a data base study following approval by the local ethics committee (S-198_2019/2024). In adherence with the ethics committee, an additional/specific consent to participate of parents or legal guardians for minors (younger than the age of 16) was not necessary, as this work was conducted as a database study.

### Study population

Twenty individuals (9 females, 11 males) exclusively with unilateral CP (GMFCS [33] level I-II) and a mean age of 12.9 ± 4.1 years [8–20 years] at the time of the index surgery matched our inclusion criteria:

- patients with unilateral CP exclusively
- no Botulinumtoxin–A injections within the last six months
- no previous surgery of the lower limbs (other than derotation osteotomy)
- increased femoral and/or tibial torsion
- exclusively femoral (and tibial) derotation osteotomy [not as part of (bony) SEMLS; e.g. no supracondylar extension osteotomy, no proximal femoral varus osteotomy]
- consecutive preoperative and postoperative/follow-up (IGA)

### 3D-instrumented gait analysis (IGA)

The first and preoperative IGA (T0) was performed from 2004 to 2023 using a 120-Hz 9-camera system (Vicon, Oxford Metrics, Oxford, UK) and two piezoelectric force plates (Kistler, Winterthur, Switzerland). Reflective markers were applied to bony landmarks according to the Plug-In Gait lower body model and protocol [34, 35]. In this procedure, the knee axis was determined by the examiner via a knee alignment device. Foot progression, describing the orientation of the foot’s long axis in relation to the gait direction, has been chosen instead of ankle rotation, since this parameter is given more clinical importance. The participants walked a seven-meter walkway barefoot and at a self-selected speed. After a mean follow-up of 19.6 ± 9.3 months following DO a second postoperative IGA (T1) was performed using the identical protocol and set-up.

### Surgical procedure

Once the indication for derotation osteotomy due to malalignment of the lower limb was made, the patients were scheduled for surgery. FDO was performed through a lateral approach to the distal femur. When necessary, tibial DO was also performed distally through a medial approach. The amount of derotation was determined individually considering the preoperative IGA and anatomical conditions (femoral torsion; tibial torsion/foot thigh angle). For internal fixation of the DO 4.5 or 3.5 Locking Compression Plates were used. The postoperative aftercare included regular physical therapy, a gradual increase in range of motion (ROM), and a gradual increase in weight-bearing after 4–6 weeks of non-weight bearing and after the first radiographic follow-up.

### Data analysis

Gait features were processed via commercial software by Vicon (Vicon Nexus 2.12, Oxford Metrics, Oxford, UK) using the Plug-In Gait model averaging at least five strides [34–36]. For visual inspection of stride-to-stride consistency as well as time normalization of gait data to the gait cycle (GC in %), lab-specific software codes were used on the basis of Matlab R2018b (MathWorks, Natick, MA, USA). Motion data were derived for the involved limbs during the whole gait cycle (GC in %).

The following gait features were considered and further analyzed:

- pelvic rotation, pelvic and trunk obliquity
- hip rotation, hip abduction, internal hip abduction moment and hip abduction power
- knee rotation
- foot progression

The reference data was derived from a group of typically developing individuals (TD) from our gait laboratory data base. The TD reference group of 26 participants (52 limbs) with a mean age of 13.1 ± 3.9 years.

### Statistical analysis

Data were structured using Microsoft Excel (Microsoft, Redmond, WA, USA). and analyzed using Matlab R2018b (MathWorks, Natick, MA, USA) For descriptive statistics the mean, the standard deviation (SD) and range were calculated. For the comparative statistics of demographics and discrete parameters a t-test for independent samples (between GMFCS levels) and for dependent samples (between time points) was used. For comparative continuous statistics alpha-corrected one-dimensional statistical parametric mapping (SPM) was performed with ANOVA-1D throughout the entire gait cycle using custom scripts in Matlab based on previous own work and on the work of Pataky et al. [37–40].

The level of significance was set at *p* < 0.05.

## Results

### Study population/patient characteristics

The demographic characteristics of the total cohort and the subgroups according to the GMFCS levels are displayed in Table 1. Importantly, there were no statistically significant differences between the subgroups regarding age, mean follow-up or the amount of femoral derotation. Also, as shown in Table 1, the amount of tibial derotation (when necessary) was found to be significantly different. In total, femoral derotation osteotomy was performed in 17 out of 20, tibial derotation osteotomy in 8 out of 20 individuals. Combined femoral and tibial derotation osteotomy was performed in 5 out of 20 individuals.

**Table 1:** Demographics and descriptive statistics of the total study population and subgroups including the corresponding *p*-values

	*n*	age years ± SD [min – max]	sex ratio f: m	IGA before DO months ± SD [min – max]	follow-up months ± SD [min – max]	femoral DO °±SD [min – max]	tibial DO °±SD [min – max]
total	20	12.9 ± 4.1 [8–20]	9:11	3.9 ± 2.9 [1–10]	19.6 ± 9.3 [12–39]	20.3 ± 9.9 [0–35]	10.0 ± 12.0 [0–30]
GMFCS I	11	12.3 ± 3.5 [8–18]	5:8	4.4 ± 3.6 [1–10]	19.6 ± 9.2 [12–39]	19.1 ± 9.7 [0–30]	3.6 ± 7.7 [0–20]
GMFCS II	9	13.7 ± 4.7 [8–20]	6:3	3.3 ± 2.1 [1–7]	19.7 ± 9.5 [12–36]	21.9 ± 9.9 [0–35]	18.8 ± 11.4 [0–30]
*p*-values (between levels)		*0.461*		*0.411*	*0.978*	*0.572*	*0.005*

### Results of the 3D-instrumented gait analyses (IGA)

**Fig. 1:**
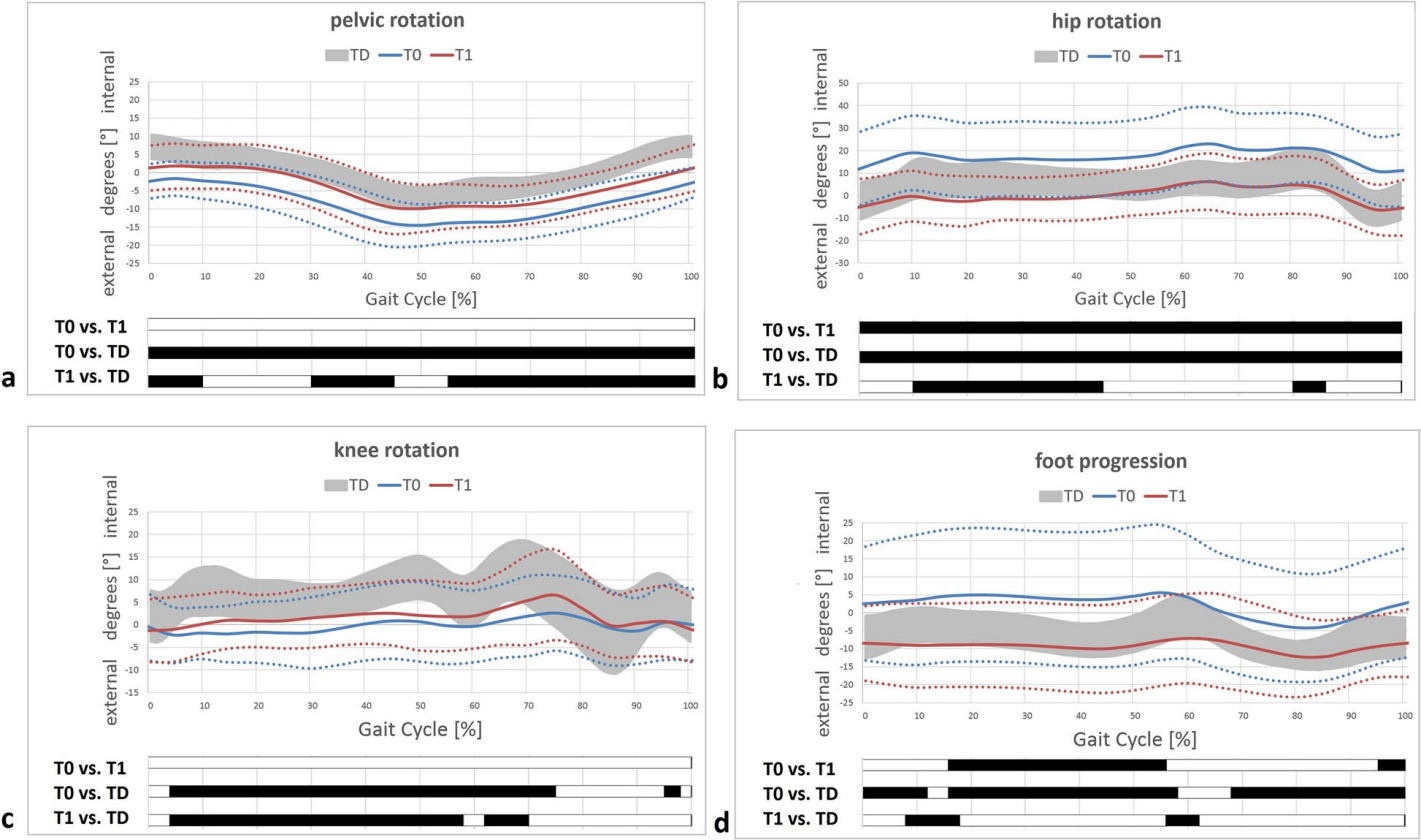
Transversal gait features of the total study population. Pelvic kinematics (**a**); hip kinematics (**b**); knee kinematics (**c**) and foot kinematics (**d**). T0/T1 preoperative and postoperative time points of IGA. TD group (typically developing individuals) of the gait laboratory data-base; grey area representing mean±1SD. Black bars (results of SPM) indicate significant differences during the corresponding parts (in %) of the gait cycle

**Fig. 2:**
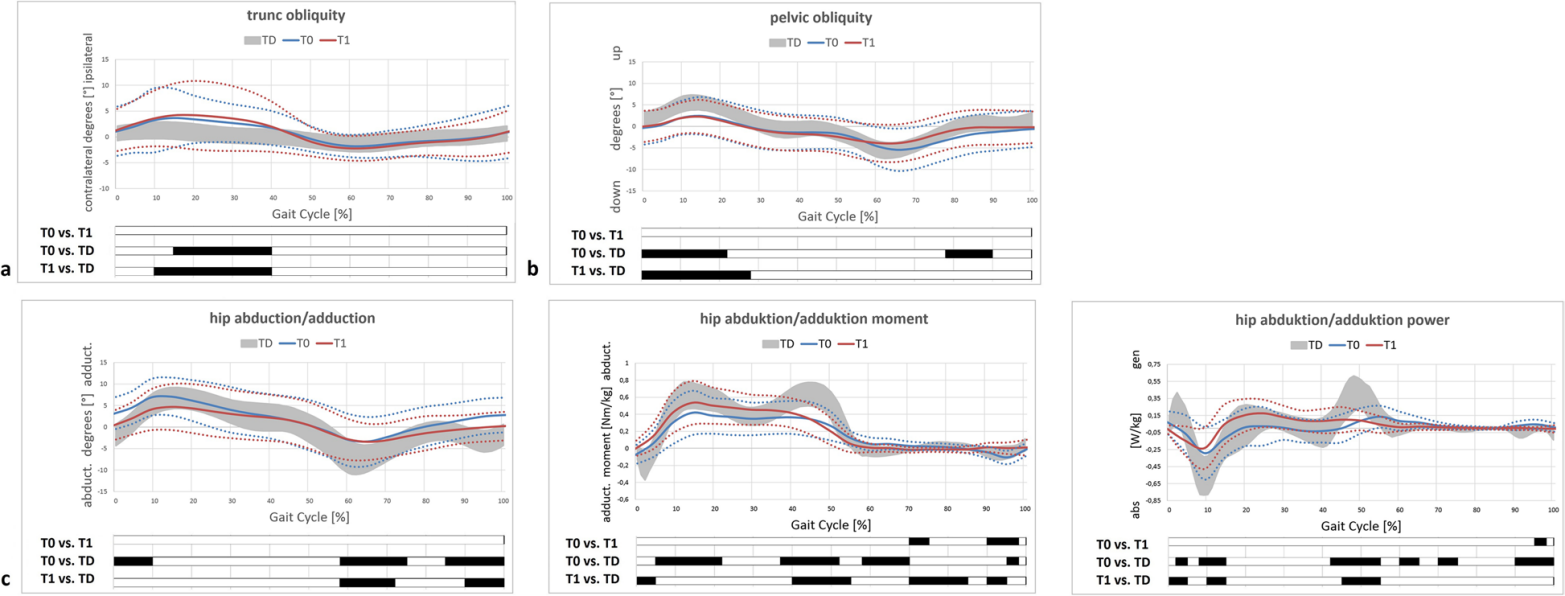
Coronal gait features of the total study population. Trunk kinematics (**a**); pelvic kinematics (**b**) and hip features (**c**). T0/T1 preoperative and postoperative time points of IGA. TD group (typically developing individuals) of the gait laboratory data-base; grey area representing mean±1SD. Black bars (results of SPM) indicate significant differences during the corresponding parts (in %) of the gait cycle

**Fig. 3:**
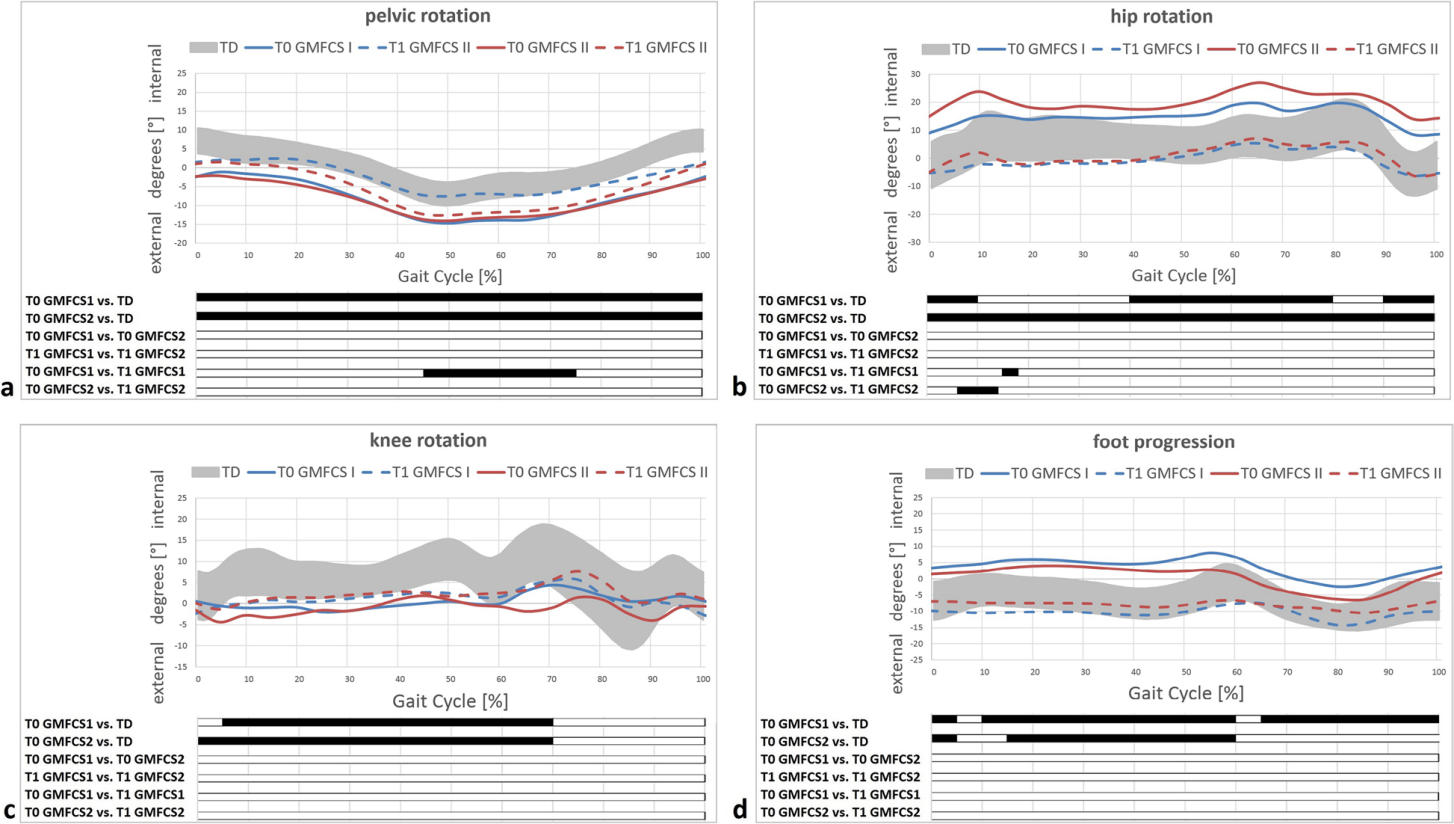
GMFCS level-specific transversal gait features. Pelvic kinematics (**a**); hip kinematics (**b**); knee kinematics (**c**) and foot kinematics (**d**). T0/T1 preoperative and postoperative time points of IGA. TD group (typically developing individuals) of the gait laboratory data-base; grey area representing mean±1SD. Black bars (results of SPM) indicate significant differences during the corresponding parts (in %) of the gait cycle

**Fig. 4:**
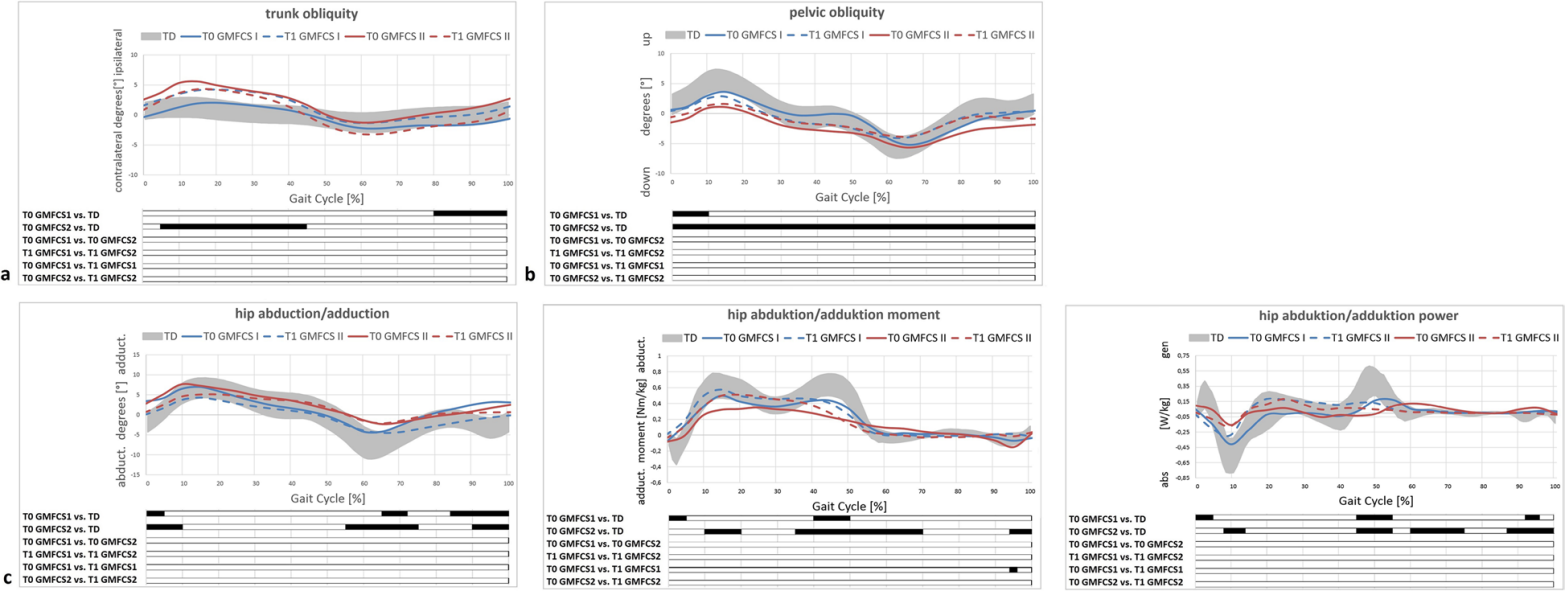
GMFCS level-specific coronal gait features. Trunk kinematics (**a**); pelvic kinematics (**b**) and hip features (**c**). T0/T1 preoperative and postoperative time points of IGA. TD group (typically developing individuals) of the gait laboratory data-base; grey area representing mean±1SD. Black bars (results of SPM) indicate significant differences during the corresponding parts (in %) of the gait cycle

Table 2 and Figs. 1, 2, 3 and 4 display all measured gait features for relevant and specific phases during gait and over the course of the entire and averaged gait cycle.

**Table 2:** Discrete parameters for the different gait features including corresponding *p*-values

gait features	total	GMFCS I	GMFCS II	*p*-values (between levels)
*transversal parameters*				
T0 pelvic rotation – stance phase mean ± SD	-7.6 ± 5.3	-7.5 ± 5.4	-7.9 ± 5.3	*0.873*
T1 pelvic rotation – stance phase mean ± SD	-3.2 ± 6.4	-1.8 ± 4.9	-4.9 ± 7.5	*0.308*
* p-values (T0 vs. T1)*	*0.0001*	*0.002*	*0.102*	
T0 hip rotation – stance phase mean ± SD	16.5 ± 16.1	14.3 ± 15.2	19.1 ± 16.8	*0.528*
T1 hip rotation – stance phase mean ± SD	-0.9 ± 10.3	-1.5 ± 10.7	-0.1 ± 9.7	*0.768*
* p-values (T0 vs. T1)*	*< 0.0001*	*0.004*	*0.001*	
T0 knee rotation – stance phase mean ± SD	-1.0 ± 7.0	-0.7 ± 5.3	-1.3 ± 8.6	*0.859*
T1 knee rotation – stance phase mean ± SD	1.1 ± 6.3	0.9 ± 4.7	1.4 ± 7.8	*0.880*
* p-values (T0 vs. T1)*	*0.157*	*0.454*	*0.214*	
T0 foot progression – stance phase mean ± SD	4.2 ± 18.1	5.3 ± 19.9	3.0 ± 15.7	*0.789*
T1 foot progression – stance phase mean ± SD	-9.0 ± 11.8	-10.3 ± 14.1	-7.6 ± 7.8	*0.629*
* p-values (T0 vs. T1)*	*< 0.001*	*< 0.001*	*0.072*	
*coronal parameters*				
trunk obliquity – stance phase mean ± SD	1.8 ± 3.9	0.6 ± 0.9	3.2 ± 5.4	*0.143*
trunk obliquity – stance phase mean ± SD	2.1 ± 4.8	2.3 ± 5.8	1.8 ± 3.2	*0.829*
* p-values (T0 vs. T1)*	*0.790*	*0.375*	*0.157*	
pelvic obliquity – stance phase mean ± SD	-0.2 ± 3.9	0.7 ± 3.8	-1.5 ± 3.6	*0.229*
pelvic obliquity – stance phase mean ± SD	-0.5 ± 3.6	-0.4 ± 2.8	-0.7 ± 4.3	*0.860*
* p-values (T0 vs. T1)*	*0.661*	*0.201*	*0.388*	
hip abduction – single support phase mean ± SD	4.5 ± 4.9	3.7 ± 4.5	5.4 ± 5.1	*0.471*
hip abduction – single support phase mean ± SD	3.2 ± 5.3	2.4 ± 6.3	4.3 ± 3.5	*0.451*
* p-values (T0 vs. T1)*	*0.267*	*0.455*	*0.406*	
hip abduction moment – single support phase mean ± SD	0.37 ± 0.19	0.41 ± 0.18	0.32 ± 0.18	*0.271*
hip abduction moment – single support phase mean ± SD	0.46 ± 0.19	0.48 ± 0.23	0.44 ± 0.11	*0.715*
* p-values (T0 vs. T1)*	*0.009*	*0.257*	*0.016*	
hip abduction power – single support phase mean ± SD	-0.05 ± 0.16	-0.07 ± 0.11	-0.02 ± 0.15	*0.456*
hip abduction power – single support phase mean ± SD	0.08 ± 0.11	0.09 ± 0.09	0.06 ± 0.13	*0.644*
* p-values (T0 vs. T1)*	*0.001*	*0.008*	*0.054*	

Most remarkable findings were an improved/reduced, yet not physiological, pelvic retraction,, significantly improved internal rotation of the hip and significantly improved foot progression (Table 2, Fig. 1).

There were no differences in coronal pelvic/trunk or hip kinematics (Table 2, Fig. 2). Coronal hip moments and joint power showed significant changes postoperatively (Table 2).

When comparing the subgroups according to GMFCS levels, most evident alterations were again improved, yet not physiological, pelvic retraction, e.g. in GMFCS level I, significantly improved internal rotation of the hip and significantly improved foot progression, e.g. in GMFCS level I (Table 2, Fig. 3). The GMFCS level II subgroup showed clinical relevant, though not statistically significant, improvement of foot progression following DO (Table 2, Fig. 3).

Regarding the measured hip features, there were significant/relevant changes evident compared to the TD or between the subgroups and time points in the SPM analysis (Fig. 4), whereas significant differences in the comparison of discrete parameters were evident (Table 2). Interestingly, pelvic and trunk obliquity showed subgroup-specific differences preoperatively. Pelvic obliquity and trunk lean were more pronounced compared to the TD in the GMFCS level II subgroup. Trunk obliquity worsened clinically relevant following DO though not significant, in the level I subgroup (Table 2, Fig. 4).

## Discussion

Transverse plane malalignment due to an increased femoral torsion – and partially concomitant increased tibial torsion – is an often encountered deformity in patients with CP which negatively affects lever arm function of the hip abductors promoting Trendelenburg and Duchenne limp. It remains unclear whether DO is capable to additionally and significantly improve lever arm dysfunction, improve Duchenne limb and restore pelvic symmetry specifically in patients with unilateral CP. These factors are considered to negatively affect the outcome after DO and promote recurrence of transverse plane malalignment.

Therefore, the goal of this study was a detailed analysis of the functional outcome following DO in patients with unilateral CP using IGA including coronal gait features of pelvis and hip.

Our results suggest that femoral/tibial DO effectively improves pelvic retraction, internal hip rotation and foot progression. However, there were no relevant changes evident concerning coronal hip kinematics, indicating that DO does not improve lever arm dysfunction of the weak hip abductors in this population of unilaterally involved individuals at short-term clinically relevant. Mildly involved individuals (GMFCS level I) showed relevant but statistically not significant worsening of the trunk lean postoperatively (Table 2, Fig. 4). Despite relevant/obvious improvements regarding transverse plane kinematics in both subgroups and significant differences in the comparison of discrete parameters, the statistical analysis (SPM) did not show significant changes (Fig. 2). This might be due to an underpowered subgroup analysis. However, changes were clinically relevant and corresponding to those of the total cohort (Figs. 1 and 2). The amount of improvement corresponded well to the intraoperative amount of derotation (Table 1, Fig. 3).

Regarding the postoperative changes of transverse plane kinematics, our results are supported by the findings of other authors [18, 20, 27, 28, 32, 41]. A significant improvement in hip rotation and foot progression angel was evident postoperatively and the outcomes were maintained from short- to long-term [18, 20, 27, 28, 41]. However, long-term hip rotation was not fully corrected by the procedure [27, 28, 41]. 

Particularly concerning pelvic asymmetry, our results indicate an improved/reduced, yet not physiological, pelvic retraction following DO at short-term. However, the findings of comparable studies available in the literature are inconsistent. Some authors describe significantly improved pelvic symmetry postoperatively [18, 20, 28, 42, 43], whereas Chung et al. observed persistent or not fully corrected pelvic asymmetry after DO, which was affected/caused by the performance of the FDO, severe preoperative pelvic rotation and the postoperative internal hip rotation [44]. Additionally, Kim et al. observed that overall pelvic rotation was not changed postoperatively, but not when preoperative compensatory pelvic rotation exceeded 5° [41]. 

With regard to hip abductor function, our results indicate that hip abduction lever arm function remains insufficient in the short term, despite the correction of the causative increased femoral torsion and improved coronal hip moments and power. In individuals functioning in GMFCS level I interestingly clinically relevant but statistically not significant worsening of the trunk lean (Trendelenburg), which is considered as a compensation mechanism [9, 11, 14, 45], was evident postoperatively at short-term. In a cohort of bilaterally involved individuals, Thielen et al. observed increased frontal hip moments one year following supracondylar FDO [8]. Also in a cohort of bilaterally involved individuals, Boyer et al. found unchanged mean hip abductor moment and unchanged pelvic/trunk obliquity at short-term [32]. However, 3 years after the index surgery (mid-term follow-up) Boyer et al. observed improved hip abductor moments and concluded that this initial lack of improvement may be caused by gait compensations that unloaded the hip [32]. 

There are limitations to our study. Since this work was conducted as a retrospective database study, it is subject to inherent limitations concerning direct control over data quality. Furthermore, the relatively small sample size limits statistical power, particularly with regard to the subgroup analysis comparing GMFCS levels. Due to the short follow-up period definitive conclusions regarding the long-term efficacy of DO cannot be drawn.

To our best knowledge, this study was the first to include trunk, pelvis and coronal hip gait features (besides transverse plane kinematics) in an cohort of exclusively unilaterally involved individuals following DO. However, it remains unclear whether the cohort examined in this study would show an improvement in hip gait features in the mid-term follow-up based on the available short-term data.

## Conclusion

DO is capable to significantly and effectively improve transverse plane malalignment in patients with unilateral CP. Trendelenburg limb, as a compensatory mechanism for weak hip abductors, remains unchanged postoperatively at short-term Mid- to long-term results in this exclusively unilaterally involved cohort are necessary to conclusively assess the evolvement of pelvic and hip (coronal) gait features.

## Data Availability

The datasets supporting the conclusions of this article are included within the article.

## Abbreviations

CP: Cerebral Palsy
DO: Derotational osteotomy
FDO: Femoral derotational osteotomy
GMFCS: Gross Motor Function Classification System
IGA: 3D-instrumented gait analyses
ROM: Range of motion
SEMLS: Single event multi-level surgery
SD: Standard deviation
TD: Typically developing

## References

[1] Staheli LT, et al. Lower-extremity rotational problems in children. Normal values to guide management. J Bone Joint Surg Am. 1985;67(1):39–47. 3968103

[2] Grisch D, Dreher T. [Torsion and torsional development of the lower extremities]. Orthopade. 2019;48(6):523–30. 31089774 10.1007/s00132-019-03752-3

[3] Theologis T. Lever arm dysfunction in cerebral palsy gait. J Child Orthop. 2013;7(5):379–82. 24432098 10.1007/s11832-013-0510-y PMC3838510

[4] Riad J, Finnbogason T, Broström E. Anatomical and dynamic rotational alignment in spastic unilateral cerebral palsy. Gait Posture. 2020;81:153–8. 32738739 10.1016/j.gaitpost.2020.07.010

[5] Salazar-Torres JJ, et al. Pelvic kinematics and their relationship to gait type in hemiplegic cerebral palsy. Gait Posture. 2011;33(4):620–4. 21454079 10.1016/j.gaitpost.2011.02.004

[6] Arnold AS, Delp SL. Rotational moment arms of the medial hamstrings and adductors vary with femoral geometry and limb position: implications for the treatment of internally rotated gait. J Biomech. 2001;34(4):437–47. 11266666 10.1016/s0021-9290(00)00232-3

[7] Arnold AS, Komattu AV, Delp SL. Internal rotation gait: a compensatory mechanism to restore abduction capacity decreased by bone deformity. Dev Med Child Neurol. 1997;39(1):40–4. 9003728 10.1111/j.1469-8749.1997.tb08202.x

[8] Thielen M, et al. Supracondylar femoral rotation osteotomy affects frontal hip kinetics in children with bilateral cerebral palsy. Dev Med Child Neurol. 2019;61(3):322–8. 30255540 10.1111/dmcn.14035

[9] Krautwurst BK, et al. The influence of hip abductor weakness on frontal plane motion of the trunk and pelvis in patients with cerebral palsy. Res Dev Disabil. 2013;34(4):1198–203. 23396196 10.1016/j.ridd.2012.12.018

[10] Salami F, et al. Ankle joint dorsi-plantar flexion and recurrence after femoral de-rotation osteotomy. Gait Posture. 2021;90:221–2.

[11] Salami F, et al. What is the price for the Duchenne gait pattern in patients with cerebral palsy? Gait Posture. 2017;58:453–6. 28918356 10.1016/j.gaitpost.2017.09.006

[12] Tsitlakidis S, et al. GMFCS Level-Specific Differences in Kinematics and Joint Moments of the Involved Side in Unilateral Cerebral Palsy. J Clin Med. 2022;11(9):2556. 35566682 10.3390/jcm11092556 PMC9100606

[13] Domagalska ME, Szopa AJ, Lembert DT. A descriptive analysis of abnormal postural patterns in children with hemiplegic cerebral palsy. Med Sci Monit. 2011;17(2):CR110–6. 21278687 10.12659/MSM.881396 PMC3524706

[14] Kiernan D. The relationship of trunk kinematics and kinetics with lower limb pathology during gait in children with spastic cerebral palsy. Gait Posture. 2021;86:33–7. 33677176 10.1016/j.gaitpost.2021.02.032

[15] Kiernan D, et al. Pathological Movements of the Pelvis and Trunk During Gait in Children With Cerebral Palsy: A Cross-Sectional Study With 3-Dimensional Kinematics and Lower Lumbar Spinal Loading. Phys Ther. 2018;98(2):86–94. 29106655 10.1093/ptj/pzx113

[16] Szopa A, Domagalska-Szopa M, Czamara A. Gait pattern differences in children with unilateral cerebral palsy. Res Dev Disabil. 2014;35(10):2261–6. 24946266 10.1016/j.ridd.2014.05.020

[17] Tsitlakidis S, et al. Transversal malalignment and proximal involvement play a relevant role in unilateral cerebral palsy regardless the subtype. J Clin Med. 2022;11(16):4816. 10.3390/jcm11164816. 10.3390/jcm11164816 PMC9409971 36013051

[18] Carty CP, et al. The effect of femoral derotation osteotomy on transverse plane hip and pelvic kinematics in children with cerebral palsy: a systematic review and meta-analysis. Gait Posture. 2014;40(3):333–40. 24984692 10.1016/j.gaitpost.2014.05.066

[19] Niklasch M, et al. Factors associated with recurrence after femoral derotation osteotomy in cerebral palsy. Gait Posture. 2015;42(4):460–5. 26276696 10.1016/j.gaitpost.2015.07.059

[20] Gresits OZ, et al. Impact of femoral derotation osteotomy on gait in ambulatory children with cerebral palsy: A systematic review and meta-analysis. Braz J Phys Ther. 2025;30(1):101257. 41045782 10.1016/j.bjpt.2025.101257 PMC12519288

[21] Armand S, Decoulon G, Bonnefoy-Mazure A. Gait analysis in children with cerebral palsy. EFORT Open Rev. 2016;1(12):448–60. 28698802 10.1302/2058-5241.1.000052 PMC5489760

[22] Papageorgiou E, et al. Systematic review on gait classifications in children with cerebral palsy: An update. Gait Posture. 2019;69:209–23. 30851621 10.1016/j.gaitpost.2019.01.038

[23] Theologis T, Wright J. Is 3-D gait analysis essential? By Professor James Wright: Introduction by Mr. Tim Theologis. Gait Posture. 2015;42(3):227–9. 26298160 10.1016/j.gaitpost.2015.05.018

[24] Radler C, et al. Torsional profile versus gait analysis: consistency between the anatomic torsion and the resulting gait pattern in patients with rotational malalignment of the lower extremity. Gait Posture. 2010;32(3):405–10. 20655226 10.1016/j.gaitpost.2010.06.019

[25] Dreher T, et al. Internal rotation gait in spastic diplegia–critical considerations for the femoral derotation osteotomy. Gait Posture. 2007;26(1):25–31. 17010611 10.1016/j.gaitpost.2006.07.018

[26] Bohm H, et al. Correction of gait after derotation osteotomies in cerebral palsy: Are the effects predictable? Gait Posture. 2015;42(4):569–74. 26387820 10.1016/j.gaitpost.2015.09.003

[27] Boyer E, et al. Long-term changes in femoral anteversion and hip rotation following femoral derotational osteotomy in children with cerebral palsy. Gait Posture. 2016;50:223–8. 27653149 10.1016/j.gaitpost.2016.09.004

[28] Aminian A, et al. Spastic hemiplegic cerebral palsy and the femoral derotation osteotomy: effect at the pelvis and hip in the transverse plane during gait. J Pediatr Orthop. 2003;23(3):314–20. 12724593

[29] Dreher T, et al. Long-term outcome of femoral derotation osteotomy in children with spastic diplegia. Gait Posture. 2012;36(3):467–70. 22766044 10.1016/j.gaitpost.2012.04.017

[30] Ounpuu S, et al. Long-term outcomes of external femoral derotation osteotomies in children with cerebral palsy. Gait Posture. 2017;56:82–8. 28521149 10.1016/j.gaitpost.2017.04.029

[31] O’Sullivan R, Kiernan D. Recurrent internal hip rotation gait in cerebral palsy: Case reports of two patients. HRB Open Res. 2018;1:28. 32596628 10.12688/hrbopenres.12893.1 PMC7308961

[32] Boyer ER, Novacheck TF, Schwartz MH. Changes in hip abductor moment 3 or more years after femoral derotation osteotomy among individuals with cerebral palsy. Dev Med Child Neurol. 2017;59(9):912–8. 28660621 10.1111/dmcn.13494

[33] Palisano R, et al. Development and reliability of a system to classify gross motor function in children with cerebral palsy. Dev Med Child Neurol. 1997;39(4):214–23. 9183258 10.1111/j.1469-8749.1997.tb07414.x

[34] Kadaba MP, Ramakrishnan HK, Wootten ME. Measurement of lower extremity kinematics during level walking. J Orthop Res. 1990;8(3):383–92. 2324857 10.1002/jor.1100080310

[35] Baker R, et al. *The Conventional Gait Model - Success and Limitations*, in *Handbook of Human Motion*. Cham: Springer International Publishing; 2018. pp. 489–508.

[36] Davis RB, et al. A gait analysis data collection and reduction technique. Hum Mov Sci. 1991;10(5):575–87.

[37] Pataky TC. One-dimensional statistical parametric mapping in Python. Comput Methods Biomech Biomed Engin. 2012;15(3):295–301. 21756121 10.1080/10255842.2010.527837

[38] Pataky TC. Generalized n-dimensional biomechanical field analysis using statistical parametric mapping. J Biomech. 2010;43(10):1976–82. 20434726 10.1016/j.jbiomech.2010.03.008

[39] Pataky TC, Robinson MA, Vanrenterghem J. Vector field statistical analysis of kinematic and force trajectories. J Biomech. 2013;46(14):2394–401. 23948374 10.1016/j.jbiomech.2013.07.031

[40] Pataky TC, Vanrenterghem J, Robinson MA. Zero- vs. one-dimensional, parametric vs. non-parametric, and confidence interval vs. hypothesis testing procedures in one-dimensional biomechanical trajectory analysis. J Biomech. 2015;48(7):1277–85. 25817475 10.1016/j.jbiomech.2015.02.051

[41] Kim HY, et al. Changes in gait parameters after femoral derotational osteotomy in cerebral palsy patients with medial femoral torsion. J Pediatr Orthop B. 2018;27(3):194–9. 28537994 10.1097/BPB.0000000000000467 PMC5895112

[42] Saraph V, et al. Effect of derotation osteotomy of the femur on hip and pelvis rotations in hemiplegic and diplegic children. J Pediatr Orthop B. 2002;11(2):159–66. 11943992 10.1097/00009957-200204000-00014

[43] Hara R, et al. Predictors of changes in pelvic rotation after surgery with or without femoral derotational osteotomy in ambulatory children with cerebral palsy. Bioengineering (Basel). 2023;10(10):1214. 10.3390/bioengineering10101214. 10.3390/bioengineering10101214 PMC10604869 37892944

[44] Chung CY, et al. Residual pelvic rotation after single-event multilevel surgery in spastic hemiplegia. J Bone Joint Surg Br. 2008;90(9):1234–8. 18757966 10.1302/0301-620X.90B9.20618

[45] Heyrman L, et al. Altered trunk movements during gait in children with spastic diplegia: compensatory or underlying trunk control deficit? Res Dev Disabil. 2014;35(9):2044–52. 24864057 10.1016/j.ridd.2014.04.031

